# The Role of Retinoic Acid in Establishing the Early Limb Bud

**DOI:** 10.3390/biom10020312

**Published:** 2020-02-17

**Authors:** Eleanor Feneck, Malcolm Logan

**Affiliations:** Randall Centre for Cell and Molecular Biophysics, King’s College London, Guy’s Campus, London SE1 1UL, UK; eleanor.feneck@kcl.ac.uk

**Keywords:** retinoic acid, limb bud, *Tbx5*, limb initiation

## Abstract

Retinoic acid (RA) was one of the first molecules in the modern era of experimental embryology to be shown capable of generating profound effects on limb development. In this review, we focus on the earliest events of limb development and specifically on the role of RA in establishing the domain of cells that will go on to form the limb itself. Although there is some consensus on the role of RA during the earliest stages of limb formation, some controversy remains on the mechanism of RA action and the requirement for RA signaling in forming the hindlimb buds.

## 1. Introduction 

Application of retinoic acid (RA) to the regenerating axolotl limb can re-specify cells so that segments of the limb that normally form in series (the arm, forearm, and hand) are duplicated [1] For example, following application of RA, cells of the regenerating limb stump can be coerced into forming an additional humerus, radius/ulna, and hand. Furthermore, this effect is dependent on the concentration of RA applied; higher doses produce the most complete duplication, while intermediate doses produce additional radius/ulna and hands, and the lowest doses produce only additional wrist elements [2]. RA was therefore one of the first molecules identified to fit the criteria of being a morphogen, since it was able to generate different cell fates depending on the concentration the responding cells were exposed to. At the same time, a role for RA was also proposed in producing the array of digits in the hand. Classical embryology experiments in chicks have previously identified a region of cells in the posterior of the limb bud, named the zone of polarizing activity (ZPA), due to its observed ability to generate a mirror symmetrical array of digits if grafted to the anterior of a host limb bud. Application of RA to the anterior region of the chick limb bud is capable of mimicking the activity of ZPA cells and generating a duplicated, mirror symmetrical series of limb digits [3]. The types of digits produced was similarly RA concentration-dependent. However, subsequently, it was shown that RA is not the endogenous ZPA signal and rather the explanation for the observed digit duplications was that application of RA induces ectopic expression of sonic hedgehog (Shh), the secreted signaling molecule that is expressed by ZPA cells and believed to act as a morphogen to specify the pattern of digits across the anterior-posterior axis of the distal limb [4,5]. The proximalizing action of RA during amphibian limb regeneration is consistent with proposed roles of RA in establishing the proximal-distal axis of the limb. In this model, opposing RA and fibroblast growth factor (FGF) signals produced from proximal and distal limb territories, respectively, control the elaboration of proximal-distal elements [6,7]. However, this model has been disputed [8]. 

RA is also required at the earliest stages of limb formation for the initial establishment of the limb bud. Early experimental evidence in chicks demonstrated that local administration of disulphiram, a chemical that blocks RA synthesis, causes a disruption in limb bud formation [9]. Placement of filter paper soaked in this reagent on top of, or adjacent to, the limb-forming lateral plate mesoderm at stages just prior to limb bud formation (around HH st 14) is sufficient to block forelimb and hindlimb formation. We review more recent studies that have tested the action of RA signaling in both forelimb and hindlimb formation and discuss, in some instances, the contrasting interpretations of these results.

## 2. Limb Bud Induction, Initiation, and Outgrowth

During embryonic development, the limbs are first morphologically evident as thickenings of the lateral plate mesoderm (LPM) that form pairs of budding outgrowths, the limb buds, on either side of the embryo flank. This process recruits precursor cells from specific regions of the LPM that will form all of the limb tissue derivatives except muscle. Muscle fibers are produced from myogenic precursors that migrate from the hypaxial myotomal compartment of the adjacent somites into the limb buds. Once this cohort of limb precursors is established, continued development of the limb bud is controlled intrinsically by growth factors and patterning molecules secreted from conserved signaling centers within the limb bud itself. The apical ectodermal ridge (AER) patterns the proximal-to-distal (shoulder to digit tip) axis and secretes fibroblast growth factor signals, whilst the anterior-to-posterior axis (thumb to little finger) is patterned by the secreted morphogen, Shh, produced by cells of the ZPA [10,11]. Continued growth of the limb bud relies on establishing a positive feedback loop of FGF signaling [12]. This is achieved by activating *Fgf10* expression in the LPM and the secreted protein activating *Fgf8* expression in the AER at the distal extreme of the overlying ectoderm. *Fgf8* secreted by the AER signals back to cells of the underlying mesenchyme to maintain the proliferative status of the limb mesenchyme and positively regulates expression of *Fgf10* [13,14,15]. In amniotes, the key initiators in establishing this positive loop of FGF signaling are the transcription factors, *Tbx5* and *Tbx4*, which are believed to directly regulate *Fgf10* expression in the developing forelimb and hindlimb mesenchyme, respectively [16]. In zebrafish, the situation is a little different as *Tbx5* activates transcription of *Fgf24* (which is not found in amniotes), which in turn activates *Fgf10* in the fin-forming mesenchyme [17,18,19]. The importance of these transcription factors has been demonstrated in deletion studies, where limb bud outgrowth is disrupted if either gene is ‘knocked-out’ [19,20,21,22,23]. While these studies demonstrate a requirement for *Tbx* gene input to establish the limb buds, recent evidence has revealed that *Tbx* expression is not sufficient for limb bud initiation and, instead, other inputs, including RA, are essential for forelimb and hindlimb-bud outgrowth [24]. In contrast, other studies in mice have proposed that RA is not required for hindlimb bud formation [8,25]. We review the different studies that have unveiled the instructive role of RA in the developing limb bud and discuss the different interpretation of these results.

## 3. *Tbx5* and *Tbx4* Are Not Sufficient for Limb Bud Induction

The transcription factors *Tbx5* and *Tbx4* act upstream of *Fgf10* to initiate limb bud outgrowth [19,20,22,26] and establish this domain of FGF signaling as both necessary and sufficient for limb formation. Application of a source of FGF to the embryo flank can induce the formation of an ectopic limb [27] and deletion of the *Fgf10* gene inhibits limb formation [15]. It could be predicted, therefore, that the action of *Tbx5* alone to activate *Fgf10* expression would be sufficient to initiate forelimb formation. However, a recent study has demonstrated that establishing *Tbx5* (forelimb) and *Tbx4* (hindlimb) expression domains is not sufficient for limb bud outgrowth [24]. 

Early studies in avian embryos demonstrated that an inductive signal from the paraxial mesoderm is required to trigger limb bud formation from the lateral plate mesoderm. Impermeable barriers placed between the somites (paraxial mesoderm) and the adjacent lateral plate mesoderm block forelimb and hindlimb bud formation [28,29]. In contrast, when a permeable barrier is used, limbs form with a normal morphology, although smaller in size, indicating that axial tissues are the source of a secreted signal. Using the same experimental approach with impermeable barriers, but now testing if expression of either *Tbx4/5* or *Fgf10* and *Fgf8* is initiated, it is demonstrated that neither expression of *Fgf10* in the LPM nor *Fgf8* in the overlying ectoderm are established in the forelimb forming region after barrier placement. Following this operation, *Tbx5* is expressed normally, which demonstrates that *Tbx5* expression is not sufficient to initiate forelimb bud outgrowth [24]. Equivalent results were observed following barrier placement at hindlimb levels. A *Tbx4* expression domain is established in the appropriate hindlimb-forming domain, but neither *Fgf10* nor *Fgf8* are expressed, and a hindlimb fails to form. These results demonstrate that neither *Tbx5* nor *Tbx4* expression alone is adequate to establish *Fgf10* in the limb forming territories of the lateral plate mesoderm and suggest that additional signals from axial tissues are essential and act in combination with *Tbx5* or *Tbx4* to activate *Fgf10* expression to generate both forelimb and hindlimb bud outgrowth, respectively. 

## 4. Retinoic Acid is Required for Limb Induction and Initiation

Again, in early avian studies, somites were shown to induce ectopic limb buds from the forelimb and hindlimb, forming lateral plate mesoderm explants when grafted to a non-limb region [30,31], suggesting that somites could be an important source of the secreted, inductive signal. Many subsequent studies have been instrumental in identifying RA as the inductive signal from the paraxial mesoderm that initiates limb bud outgrowth. 

Limb bud formation is blocked when the synthesis of RA is chemically inhibited in chick embryos [9]. For zebrafish and mouse embryos mutant for *retinaldehyde dehydrogenase-2* (*Raldh2*), the gene encoding a key synthetic enzyme is responsible for the majority of RA production during embryonic development, which show a failure of pectoral fin and forelimb development, respectively [32,33,34]. In zebrafish, genetic ablation of *Raldh2* in somitic mesoderm leads to a reduction of *Tbx5* expression in the fin-forming region, while treatment of similar mutant embryos with exogenous RA can reverse this effect. Furthermore, pectoral fin bud initiation is restored in mutant *Raldh2* zebrafish when grafts of wild-type somites are placed adjacent to the fin-forming region [35]. This result suggests that RA expressed from the somitic mesoderm is important to establish *Tbx5* expression in the future limb forming region [35]. 

Experiments using impermeable barriers placed between somites and lateral plate mesoderm to block limb formation have distinguished two phases of RA input in early limb formation ([24]. To recap, when a barrier is placed just prior to limb bud formation, no limb bud forms but *Tbx5* and *Tbx4* expression is established in the forelimb and hindlimb regions, respectively. If the same operation is carried out, and, in addition, a RA-soaked bead is applied to the cells of the forelimb or hindlimb-forming region, *Fgf10* expression and limb bud formation is rescued. This suggests that the RA signal emanating from the somites, which is blocked by the barrier, normally acts in combination with *Tbx* factors to activate *Fgf10* expression. When barriers are placed between axial tissues and the prospective limb-forming regions at much earlier stages, activation of *Tbx5* or *Tbx4* expression is blocked, which is consistent with RA having an earlier role in establishing expression of these *Tbx* genes in their respective domains [24], consistent with early effects seen in zebrafish and mouse *Raldh2* mutants described above.

Analysis of a putative forelimb regulatory element of the mouse *Tbx5* gene supports multiple signaling inputs in establishing its expression in the forelimb-forming region. A series of studies employing transient transgenics has mapped a core regulatory element within intron 2 of *Tbx5* that is capable of recapitulating the forelimb expression domain of the gene [36,37]. This element contains RARE, TCF/LEF (Wnt/B-catenin), and Hox binding sites, and the combination of these inputs can explain the regionalized restriction of *Tbx5* expression to the forelimb-forming region [24]. Mutation of either RARE or TCF/LEF sites disrupts activity, suggesting that RA and Wnt signaling are required to positively regulate expression. Downregulation of Wnt reduces *Tbx5* expression [19,24,38] consistent with Wnt/ß-catenin signals acting upstream of *Tbx5*. Wnt2 protein is expressed within the lateral plate mesoderm at E8.5 and is therefore a potential ligand to establish ß-catenin signaling. A combination of positive and negative regulation from 3’ and 5’ Hox genes, respectively, expressed in nested patterns along the rostral-caudal length of the lateral plate mesoderm ensure expression is restricted to the appropriate axial level.

However, a recent study using CRISPR/Cas9 technology to delete the intron 2 sequence specifically indicates that regulatory elements within this region are not required to establish *Tbx5* expression in the forelimb bud and that forelimbs can form normally in the absence of this element [39]. This study also used the same deletion strategy to test a conserved *Tbx5* regulatory element uncovered by phylogenetic foot printing [40]. Neither site, either singly or in combination, is essential for the establishment of *Tbx5* expression and further limb formation in mice. The possible role of these elements in the normal establishment of *Tbx5* expression therefore remains unclear and identifying alternative regulatory elements that control expression of this gene, which plays such a pivotal role in forelimb initiation, is necessary.

**Figure 1:**
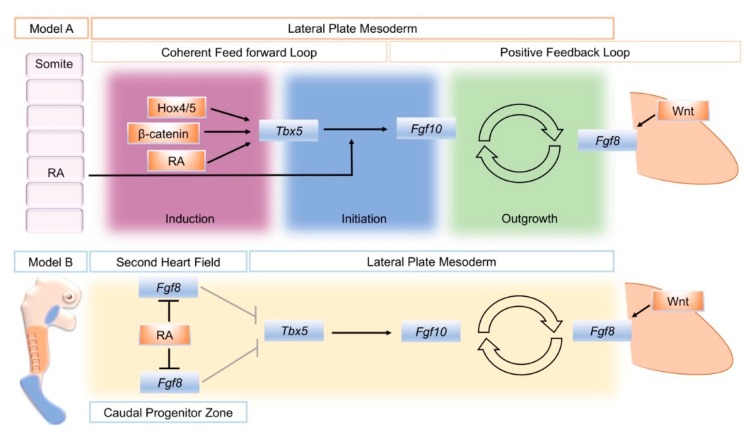
Schematic summary of two different models to explain the action of retinoic acid (RA) to establish the limb bud. Model A distinguishes three phases of limb bud development; induction, initiation, and outgrowth. RA, Wnt/ß-catenin signaling, and spatially restricted Hox genes combine to activate *Tbx5* in a restricted region of the lateral plate mesoderm that will become the forelimb-forming region (Induction). In a coherent feed-forward loop, RA also combines with RA to activate fibroblast growth factor 10 (*Fgf10)* expression in the forming limb mesenchyme (Initiation). *Fgf10* then successively activates *Fgf8* in the overlying ectoderm, and a positive feedback loop of FGF signaling is established that sustains limb bud outgrowth (Outgrowth). An alternative, model B: Mutual antagonism between retinoic acid (RA) and fibroblast growth factor 8 (*Fgf8*) enables *Tbx5* expression to be established in the future forelimb-forming region of the lateral plate mesoderm. At stages just prior to forelimb bud formation in the mouse, (E8.5) RA (orange) produced in the trunk region restricts *Fgf8* expression (blue) to the more rostrally located, second heart field and the caudal progenitor zone, de-repressing the repressive effect of *Fgf8* on *Tbx5* expression (shown by gray arrows). Model A schematic is derived from [24], Figure 1. Model B is derived from [8], Figure 3.

A model that attempts to unify data from several sources divides the establishment of the limb bud into three phases: induction, initiation, and outgrowth. Initially, a coherent feed-forward loop [41] is set-up through the action of RA and *Tbx* genes. Then, a positive feedback loop of FGF signaling between the lateral plate mesoderm and the overlying, distal ectoderm is established that sustains limb bud outgrowth (Figure 1, Model A) [24]. During the induction phase, *Raldh2* expression in axial tissue produces RA locally that acts on adjacent lateral plate mesoderm in combination with intrinsically expressed *ß-catenin* and 3’ *Hox* genes to activate *Tbx5* expression. In a coherent feed-forward loop, RA and Tbx5 together induce *Fgf10* expression. *Fgf10* produced in the limb mesenchyme (with input from Wnt signaling) subsequently induces *Fgf8* in the overlying ectoderm. This establishes the *Fgf10*–*Fgf8* positive feedback loop that drives limb bud outgrowth. 

## 5. An Alternative Model for RA Action in Forelimb Initiation

Gene deletion studies in mice targeting RA biosynthetic enzymes, retinol dehydrogenases (RDH), and retinaldehyde dehydrogenases (RALDH) have demonstrated the importance of RA signaling, and of these, the *Raldh2* and *RDH10* mutants have the most profound limb phenotypes. Deletion of *Raldh2* in mice disrupts forelimb bud formation at early stages, and no *Tbx5* expression is detected in the forelimb-forming region. As a result of the essential role *Raldh2* plays in generating the RA required for the formation of many other embryonic tissues and organs, this mutation is lethal, and the severely abnormal embryos die around E8.5–E8.75 [34]. As embryo lethality in the *Raldh2* mutants occurs prior to hindlimb formation, the role of RA in hindlimb formation cannot be deduced from this model. To attempt to circumvent the embryonic lethality, RA was administered maternally for a limited time period, which prolongs embryonic development and generates mutant embryos with stunted forelimbs, disrupted Shh, and *Fgf8* expression domains and normal hindlimb buds [25,34,42]. A similar phenotype was produced using the same maternal RA administration regime in *Raldh2/Raldh3* compound mutants [25]. The observations of the rescued *Raldh* mutant embryos led to the conclusion that RA is only required for forelimb bud formation and is not required in the hindlimb forming lateral plate mesoderm. In addition, *Rdh10* mutants have stunted forelimbs and normal hindlimbs [12,43,44]. Extensive steps were taken using both mutant lines to attempt to confirm the local reduction of RA activity and rule out the possibility that the maternal administration of RA was the explanation for hindlimb formation being able to proceed in the treated embryos. This included using a RARE-lacZ reporter that identified no RA signaling in the hindlimb bud and the lateral plate mesoderm in the RA-rescued mutant limbs [8]. 

The close association between the distribution of RA activity, the restriction of *Fgf8* expression to two domains in the second heart field and caudal end of the embryo, and the onset of *Tbx5* expression has provided the basis of a model for how RA could regulate the establishment of *Tbx5* in the forelimb-forming region (Figure 1, Model B). Prior to limb formation, RA is distributed throughout the forelimb-forming territory, where it acts to restrict *Fgf8* expression to the heart and more caudal domains and thereby enables activation of *Tbx5*. In RA signaling mutants, the reduction or absence of RA activity leads to a caudal expansion of cardiac *Fgf8* expression and rostral expansion of *Fgf8*, which blocks or severely reduces activation of *Tbx5* [8,45].

## 6. Concluding Remarks

The final steps of RA biosynthesis involve the oxidation of retinol to retinaldehyde, which can be catalyzed by several different alcohol dehydrogenase enzymes and a final oxidation of retinaldehyde to RA, catalyzed by retinaldehyde dehydrogenase. *Raldh2* is a dominant player in RA generation in the early embryo, given the profound defects seen in *Raldh2* mutants. *Rdh10* mutants noticeably have a milder phenotype than *Raldh2* nulls, indicating that there must be other pathways to produce the retinaldehyde substrate for *Raldh* in the absence of *Rdh10*. The defects found in *Rdh10* mutants therefore do not represent a complete state of RA deficiency [46]. A volume of data supports an active role for RA in forelimb and hindlimb formation, with the creation of an embryonic environment devoid of all RA, proving a challenge for the scientific community. Given the multiple enzymatic pathways that can generate RA, their broad distribution throughout the developing embryo and the ability of RA to diffuse, employing a gene deletion strategy that can entirely rule out the possibility that some RA will be produced that could influence cells in the limb-forming regions, is challenging. Furthermore, the wide domains of the embryo that need to be targeted to disrupt RA production produce additional confounding abnormalities and complicate analysis. Maternal administration of RA to mutants of RA synthetic enzymes, despite stringent controls, raises the possibility that structures have formed due to the influence of the exogenous RA source.

Genetic loss of function studies in mice are a powerful method to test gene requirement for a chosen developmental process, but their application and interpretation is not always simple. Analysis of the function of RA in early limb formation is a good example. RA is produced and can diffuse across a large area of the developing embryo and is involved in multiple events across a broad range of stages. RA synthesis is regulated by a series of biosynthetic enzymes and signals through multiple receptors. Together, this makes genetic dissection of RA roles quite challenging. Genetic deletion studies, misexpression studies, and embryological manipulations all have their place in ultimately teasing apart the myriad roles of RA in embryonic development.
